# Incidence of solid tumors in polycythemia vera treated with phlebotomy with or without hydroxyurea: ECLAP follow-up data

**DOI:** 10.1038/s41408-017-0038-3

**Published:** 2018-01-16

**Authors:** Arianna Ghirardi, Alessandra Carobbio, Arianna Masciulli, Tiziano Barbui

**Affiliations:** FROM Research Foundation, Papa Giovanni XXIII Hospital, Bergamo, Italy

Phlebotomy (PHL) and chemotherapy have substantially improved the current survival in polycythemia vera (PV)^1^.

The Polycythemia Vera Study Group (PVSG) 01 trial^2^ found the use of chlorambucil and, to a lesser extent 32P, to be associated with a high leukemogenic risk and solid tumors, while phlebotomy was less protective against thrombosis. Based on results of a phase II study^3^, Hydroxyurea (HU), alone or in association with PHL, was found efficacious and safe and is currently considered the first-line therapy in PV patients, but it has never been entered in controlled trials of adequate size and duration to assess its long-term safety. On the other side, scientific progresses in this field are very difficult due to the scarce feasibility of large size prospective studies and for the overall low rate of target events often appearing late in disease course. Therefore, whether the occurrence of hematologic and non-hematologic malignancies have increased by HU in comparison with PHL remains unclear. Large epidemiological studies may be of help to answer this relevant clinical question as solid tumors are described with increasing frequency in myeloproliferative neoplasms (MPN) compared to the general population and are one of the major causes of mortality^4–8^.

Based on these premises, we carried out an analysis in the cohort of 1638 PV patients who were screened for inclusion in the European Collaboration on Low-Dose Aspirin in Polycythemia Vera (ECLAP) study^9^ that enrolled all patients with new and old diagnoses of PV according to the PVSG criteria^2^. Treatment strategies had to comply with the recommendation of maintaining the hematocrit value at <0.45 and the platelet count at <400 × 10^9^/L. Clinical outcomes, including the occurrence of solid tumors, during the prospective follow-up were recorded at follow-up visits at 12, 24, 36, 48, and 60 months.

Out of 1638 screened patients, we identified 1042 patients treated with PHL (*n* = 342) alone or HU (*n* = 700) to annotate (a) the incidence of non-hematologic and hematologic malignancies (excluding all types of leukemia, carcinomas in situ, superficial bladder carcinoma, and non-melanoma skin cancers) and (b) to evaluate the prognostic factors in the two groups.

To assure comparability between the two groups, we also conducted a 1:1 propensity score (PS) matching analysis^10^ by forming matched sets of one PHL and one randomly sampled HU-treated patient who shared a similar values of PS. The PS was estimated by regressing exposure to only PHL conditionally on the baseline covariates (age at enrolment, gender, years from PV diagnosis, prior thrombosis, aspirin use, active smoking, and arterial hypertension) with a logistic model.

For 10 PHL patients, no suitable HU-treated patient (i.e., with a similar value of PS) was found to be matched.

Characteristics of patients before and after PS-matching were reported in Table 1.

**Table 1:** Patient characteristics and incidence rate of solid tumors during follow-up are presented before and after Propensity Score (PS)—matching

	Before PS-matching			After PS-matching		
	Total cohort (*n* = 1042)			1:1 random-sample matched cohort^*^ (*n *= 664)		
	PHL (*n* = 342)	HU (*n* = 700)	STD	PHL (*n* = 332)	HU (*n* = 332)	STD
*Baseline features of patients*						
Age at enrolment ≥60,* n(%)*	186 (54.4%)	532 (76.0%)	−1.02	186 (56.0%)	194 (58.0%)	0.00
Male,* n(%)*	238 (69.6%)	374 (53.4%)	0.33	228 (69.0%)	230 (69.0%)	0.00
Years from diagnosis of PV to enrolment ≥5,* n(%)*	102 (29.8%)	260 (37.1%)	0.15	102 (31.0%)	109 (33.0%)	−0.06
Prior thrombosis,* n(%)*	115 (33.6%)	285 (40.7%)	−0.15	115 (34.6%)	125 (37.7%)	0.00
High risk,* n(%)*	221 (64.6%)	588 (84.0%)	−0.91	221 (66.6%)	238 (71.7%)	0.00
Active smoking,* n(%)*	67 (19.6%)	83 (11.9%)	0.14	57 (17.2%)	59 (17.8%)	−0.07
Hypertension,* n(%)*	138 (40.4%)	286 (40.9%)	0.02	129 (38.9%)	135 (40.7%)	0.06
Diabetes mellitus,* n(%)*	25 (7.3%)	52 (7.4%)	−0.21	24 (7.2%)	25 (7.5%)	0.00
Antiplatelet use,* n(%)*	127 (37.1%)	309 (44.1%)	−0.11	125 (37.7%)	141 (42.5%)	0.12
*Follow-up*			*p*			*p*
Median total follow-up (IQR), months	29.9 (15.1, 41.0)	30.4 (21.6, 44.3)	0.073	29.9 (14.5, 41.1)	33.2 (22.8, 46.2)	0.003
Median treatment duration (IQR), months	25.8 (12.7, 37.3)	24.0 (12.0, 36.0)	0.925	26.2 (12.6, 37.4)	24.0 (14.6, 44.3)	0.139
Solid tumors	8 (2.3%)	23 (3.3%)	0.398	7 (2.1%)	12 (3.6%)	0.244
IR/100 PY (95% CI)	0.98 (0.49−1.96)	1.29 (0.86−1.94)	0.521	0.89 (0.42, 1.86)	1.35 (0.77, 2.38)	0.257

*PHL* Phlebotomies, *HU* Hydroxyurea, *PV* polycythemia vera, *STD* standardized difference, *IR* incidence rate; *PY* person-years, *IQR* interquartile range

*1 PHL patient: 1 randomly sampled HU patient in each matched subset (for 10 PHL patients, no suitable HU patient was found). Matching was done using the nearest neighbor method without replacement and with caliper of width equal to 0.2 of the pooled standard deviation of the logit of PS. STDs <0.1 indicate a good balance between treatment groups

Before PS-matching, >90% of patients were treated during follow-up only with PHL or HU; the duration of treatment exposure was similar in PHL and HU patients (median months 25.8 and 24.0 in PHL and HU, respectively). However, the two groups were not comparable as they showed a statistically significant difference in clinical characteristics: in comparison with PHL subjects, an higher proportion of HU patients was older than 60 years (*p* = 0.000), had experienced prior thrombotic events (*p* = 0.027), had a longer history of PV disease (*p* = 0.020) and received more frequently prophylaxis with low-dose aspirin (*p* = 0.031).

In 31 patients, solid tumors occurred after a median time from diagnosis of PV of 6 years (range 3.0–9.2): 28 patients developed carcinoma (bladder, breast, colon, stomach, kidney, laryngeal, lung, pancreas, and prostate) and 3 patients lymphoid malignancies (non-Hodgkin lymphoma). The incidence rate of these events, calculated by the ratio between the observed number of events and the corresponding person-years (PY), was similar in the two groups (0.98 vs. 1.29 per 100 PY; *p* = 0.521). In multivariable analysis, adjusting the estimated hazard ratio (HR) for baseline features, the risk of solid tumors in HU patients was not greater than that of PHL patients (HR 1.07, 95% CI 0.47–2.44; *p* = 0.876). As expected, age over 60 years was associated with higher risk of solid tumors (HR = 2.80, 95% CI 1.05–7.42; *p* = 0.039), while the use of aspirin was found to reduce the risk of the considered outcome (HR 0.32, 95% CI 0.13–0.77; *p* = 0.012). Due to the low number and the varied types of solid tumors, we could not demonstrate that low-dose aspirin had a largest effect on the reduction of a specific type of cancer (such as gastrointestinal) as reported in previous studies^11^.

After matching, the two groups were well balanced for all the baseline features (overall balance test: *p* = 0.964), providing a solid background for their assessment in terms of outcomes. Due to the matching process only 332 patients out of 700 of the HU group met the criteria for matching with PHL subjects (a similar PS value) and, as a consequence, only 19 solid tumors could be examined: 7 in PHL subjects (bladder, breast, kidney, lung, melanoma, myeloma, and prostate) and 12 in HU patients (bladder 1, breast 1, carcinoma of unknown origin 2, colon 2, gastric 1, kidney 1, laryngeal 2, pancreas 1, and prostate 1). As in the whole cohort, in the PS-matched analysis the incidence rate of these events was similar in the two groups (0.89 vs. 1.35 per 100 PY, *p* = 0.257). Similarly, the results of the Cox proportional-hazard model (stratified on the matched pairs), showed that, compared to the PHL group, HU patients did not show an higher risk of solid tumors (HR 1.60, 95% CI 0.52–4.89; *p* = 0.410).

This prospective study is a descriptive and comparative analysis of solid tumors occurring in the course of the two treatments currently recommended as first-line therapy of PV patients^12, 13^.

Some limitations should be admitted.

First, the ECLAP study enrolled patients in the era pre-*JAK2V617F* discovery (the last patient was enrolled in 2001); second, the period of prospective observation was relatively short so that the incidence of tumors could be underestimated; third, the low number of events could weaken the reliability of this analysis. However, we underscore that these patients were monitored prospectively in a qualified network of hematologic centers by using the same criteria of a clinical trial and in particular the observed events were validated by a panel of experts in the field; moreover, we believe that the rigorous application of a propensity score matching is expected to reduce most of the underlying limitations. Future studies are warranted to elucidate the pathophysiology that leads to the occurrence of solid tumors in MPN disorders and to explore whether MPN could also predispose patients to the development of secondary malignancies. From our analysis, the hypothesis that malignant events are related to long-term toxicity of HU use, seems to be ruled out although this finding needs confirmation. The preventing role exerted by low-dose aspirin should be highlighted and deserves future clinical and pharmacologic studies. Finally, clinical studies are warranted to better refine the target population at high risk of developing tumors in order to promote programs of active surveillance particularly in the current era of *JAK2* inhibitor drugs.
